# Data on air temperature, relative humidity, and dew point in three housing modes in a building in a hot and humid area of Douala, Cameroon

**DOI:** 10.1016/j.dib.2025.112120

**Published:** 2025-09-30

**Authors:** Thomas Janvier Matongo, Gilbert Roméo Hubert Ngock, Emmanuel Yamb, Jean Gaston Tamba

**Affiliations:** a University of Douala, University Institute of Technology, Cameroon; b University of Douala, Advanced Technical Teachers Training College, Cameroon

**Keywords:** Temperature data, Humidity data, Dew point data, Residential building, Hot and humid climate

## Abstract

The purpose of this article is to present the spatiotemporal dynamics of temperature, relative humidity and dew point in a residential building according to three dwelling modes in Douala, Cameroon. These microclimatic parameters were collected outside and inside three rooms, namely an open and unoccupied room (S1), a closed and unoccupied room (S2) and an occupied room (S3). Data collection were carried out over 365 days at one-minute intervals, resulting in a total of 525,600 records for each parameter. The data were collected using EXTECH^Ⓡ^ RHT10 thermohygrometers. It was then transferred and processed in Excel and imported into MATLAB software to represent seasonal variations. The data collected can be used in simulation software to model the thermal performance of buildings in tropical areas. They can also be used to help design walls, roofs and windows to minimise overheating during the dry season and heat loss in the cold season.

Specifications Table

**Table:** 

Subject	Engineering & Materials science
Specific subject area	Building thermal energy
Type of data	Table, Image, Figure.
Data collection	The data were collected in outdoor and indoor climatic environments at experimental facilities located in a hot and humid climate. The premises consist of three rooms: one unoccupied open room, one unoccupied closed room and one occupied room. Data collected using EXTECH RHT10 thermohygrometers; Copyright © 2014 FLIR Systems, Inc. Temperature and relative humidity data were collected at one-minute intervals over 365 days in 2019. Excel software was used to process the data, and the curves were plotted using Matlab software.
Data source location	Institution: University of Douala Country: Cameroon Latitude and longitude (and GPS coordinates, if possible) for collected samples/data: 4.0193785; 9.8006592.
Data accessibility	The data can be accessed at Mendeley data: MATONGO, THOMAS JANVIER; Ngock, Gilbert Roméo Hubert; LEOPOLD, MBA; Tamba, Jean Gaston; Yamb, Emmanuel; Madouma, romaric (2025), “Data on air temperature, relative humidity, and dew point in three housing modes in a building in a hot and humid area of Douala, Cameroon”, Mendeley Data, V1, doi: 10.17632/7dmkptsj2h.1 Graphical representations are available on Mendeley Data: NGOCK Gilbert Romeo Hubert; MATONGO, Thomas Janvier (2025), “Figures of Data on air temperature, relative humidity, and dew point in three housing modes in a building in a hot and humid area of Douala, Cameroon”, V1, doi: 10.17632/jbrpzcrvj9.1 Repository name: Mendeley Data,V1 Data identification numbers: doi: 10.17632/7dmkptsj2h.1 And: doi: 10.17632/jbrpzcrvj9.1 Direct URL to data: https://data.mendeley.com/datasets/7dmkptsj2h/1 And: https://data.mendeley.com/datasets/jbrpzcrvj9/1
Related research article	none

## 1 Value of the Data

The collection of experimental data on ambient temperature, relative humidity and dew point offers several significant benefits:

• Thermal comfort simulations
  The data collected can be integrated into simulation software to model the thermal performance of buildings in tropical areas.
• Passive cooling design
  This data can be used to design walls, roofs and windows to improve the performance of the building envelope by minimising overheating in the dry season and heat loss in the cold season.
• Understanding the climate:
  Trend analysis can be used to identify typical and extreme climatic conditions in order to plan a building's energy requirements.
• Simulation validation for tropical climates:
  Simulation results can be compared with data measured on site to verify whether the simulation correctly predicts actual conditions.

## 2 Background

Thermal discomfort and humidity in buildings have become a major nuisance in Douala, particularly in modern homes built with unconditioned concrete blocks. The severity of this problem, which has been little studied to date, varies from room to room within the same building. These differences are mainly related to the behaviour of the occupants, air circulation, air temperature and humidity. To obtain the parameters needed to better understand this problem, an initial hourly data collection was published in 2022, https://doi.org/10.1016/j.dib.2022.107906. However, it did not take into account the dew point. In this article, we add this essential parameter in the formation of humidity as well as air temperature and relative humidity, but at a reduced interval of one minute. This provides a high degree of accuracy in the use of the data.

## 3 Data Description

The data on air temperature, relative humidity and dew point presented in this paper show, on a reduced scale of one minute, the temporal evolution of the microclimatic parameters of the premises of a building in the city of Douala in Cameroon (Fig. 7). The dew point was measured directly by EXTECH^Ⓡ^ sensors, model RHT10; Copyright © 2014 FLIR Systems, Inc. ISO‐9001 certified. Dew point was calculated using the RHT10-SW programme, which is not accessible to users. Three housing modes were defined and 525,600 records of each parameter were collected over 365 days in 2019. The data collected is grouped into monthly series simultaneously in the three rooms.

- Room S1 is open to the outside environment and is uninhabited

- Room S2 is closed and is uninhabited

- Room S3 is inhabited

Each month has nine parameters

 : The outside air temperature TEX

- The relative humidity of the outside air HEX

- The dew point of the outside air DEX

- The indoor air temperature of the empty open room S1 TS1

- The relative humidity of the indoor air in the empty open room S1 HS1

- The dew point of empty open room S1 DS1

- Indoor air temperature of the empty closed room S2 TS2

- The Relative humidity of the closed empty room S2 HS2

- The dew point of the closed room S2 DS2

- The indoor air temperature in room S3, occupied TS3

- The relative humidity of indoor air in room S3, occupied HS3

- The dew point of occupied room S3 DS3

### 3.1 Graphical representation of data

In this section, we will present the spatiotemporal dynamics of the parameters for the warmest month (February) and the coldest month (August). For a complete analysis of the climatic conditions for each month of the year, please consult the following link: Mendeley Data, V1, doi: 10.17632/jbrpzcrvj9.1

**Fig. 1:**
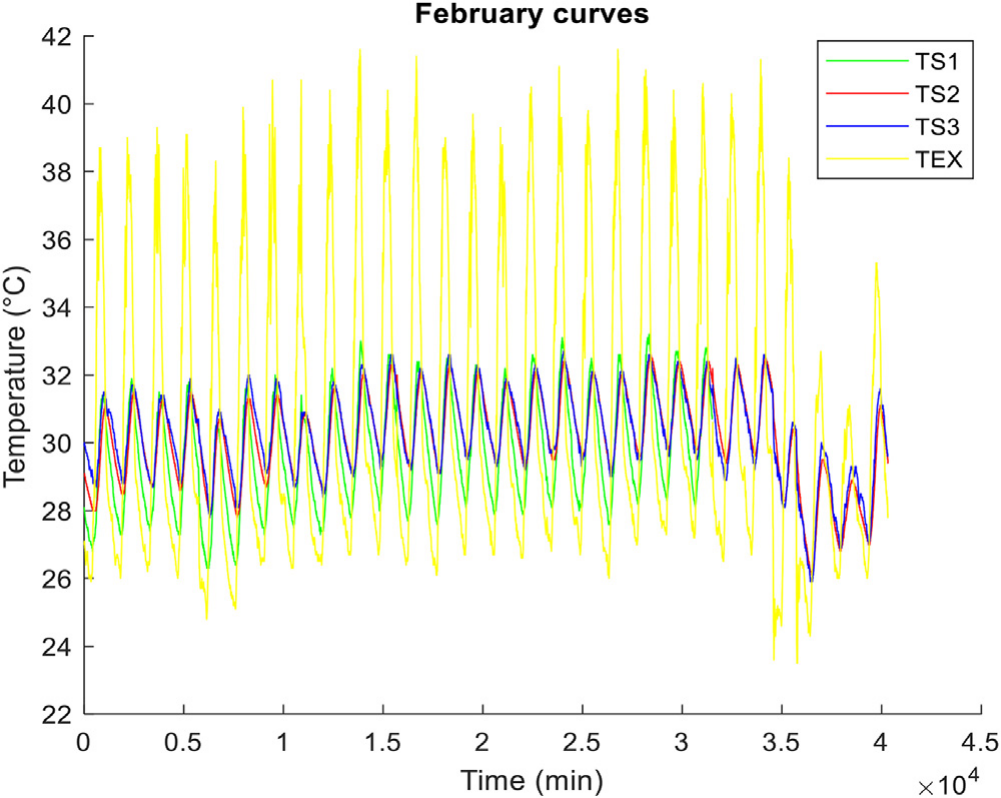
Air temperature of the hottest month.

**Fig. 2:**
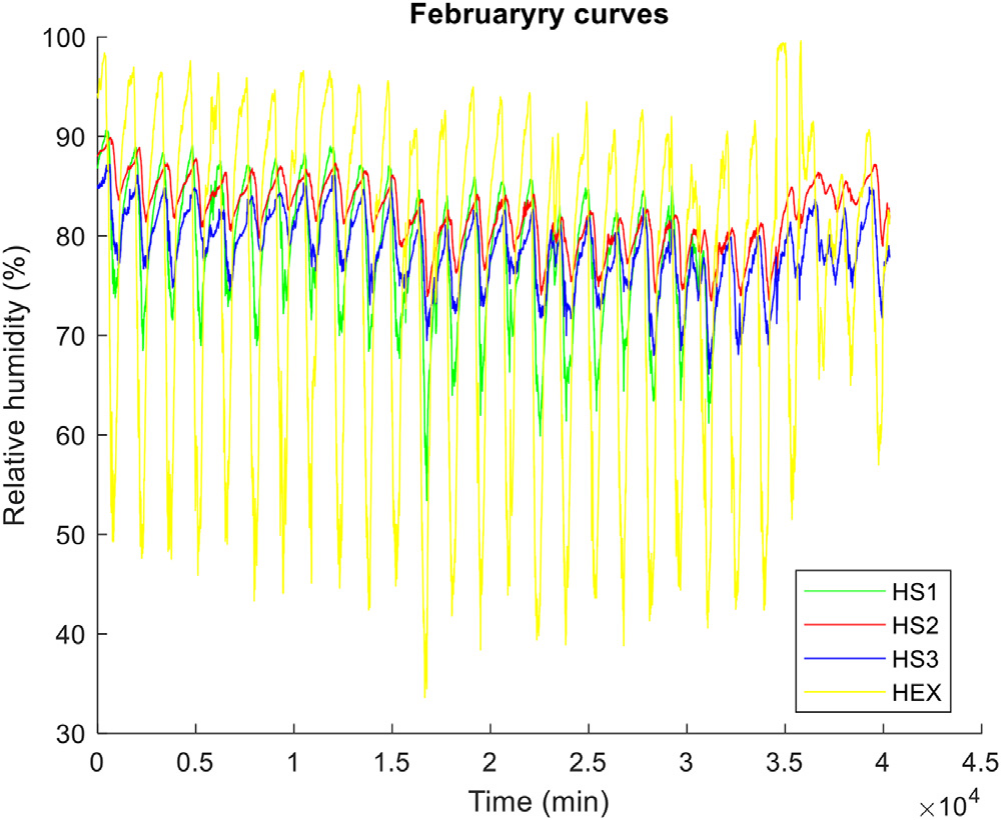
Relative humidity of the air in the hottest month.

**Fig. 3:**
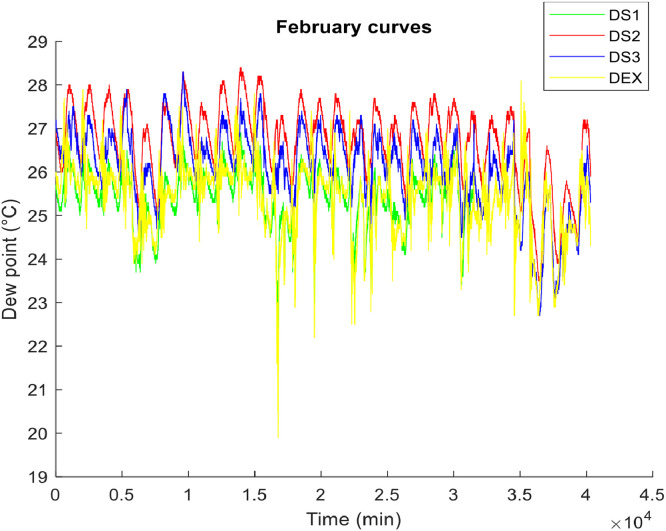
The dew points of the air in the hottest month.

**• The hottest month (** Fig. 1, Fig. 2, Fig. 3 **)**

**Fig. 4:**
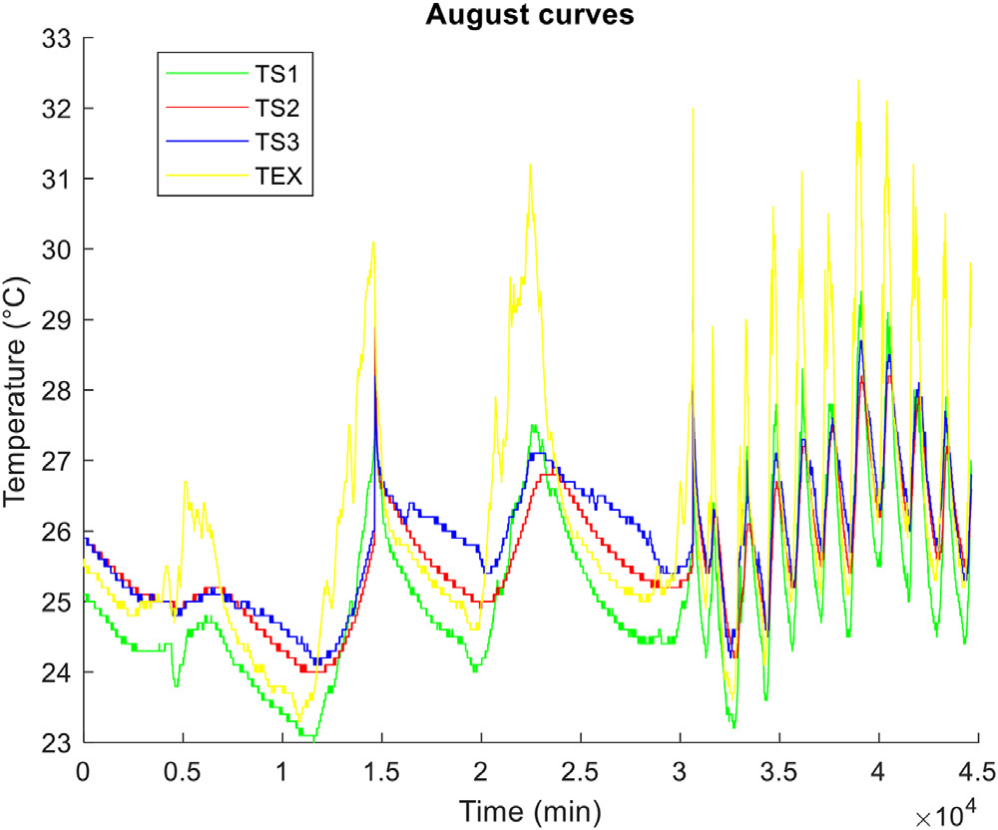
Air temperature of the coldest month.

**Fig. 5:**
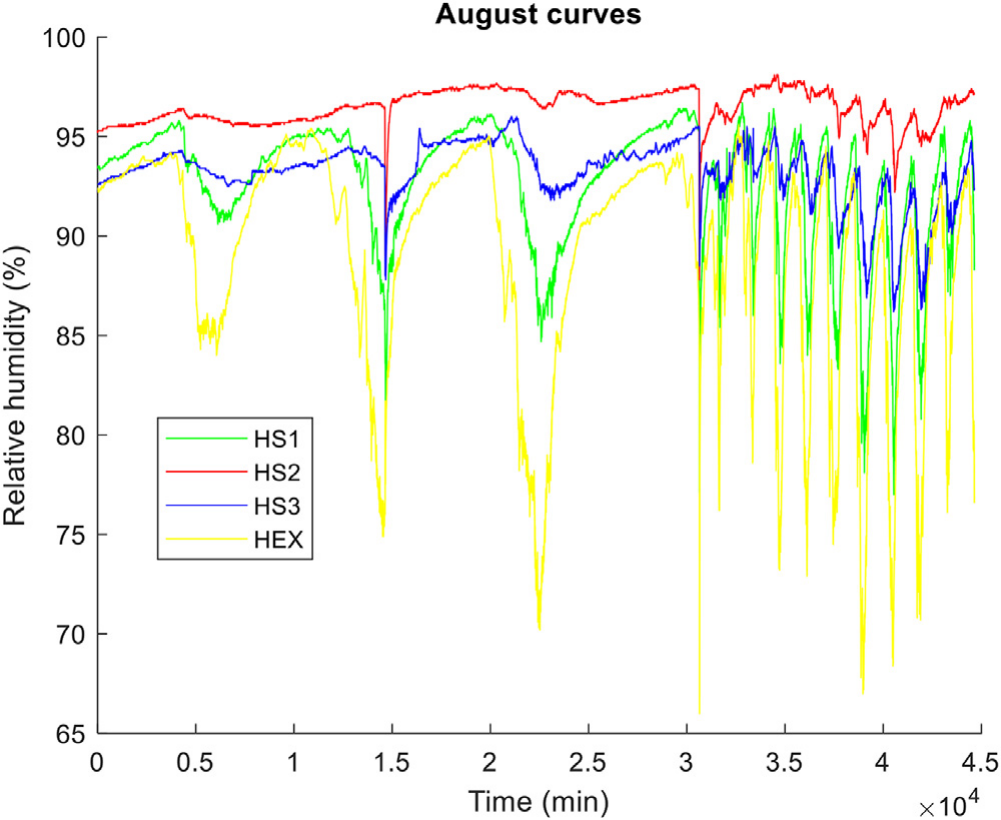
Relative humidity of the air in the coldest month.

**Fig. 6:**
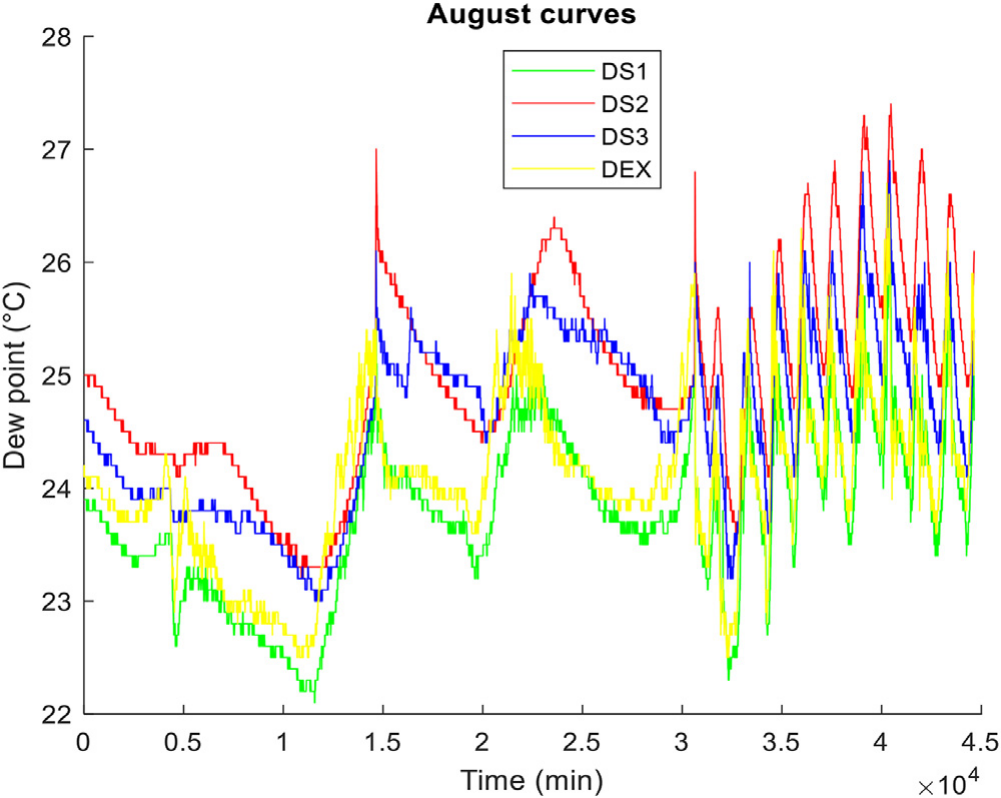
The dew points of the air in the coldest month.

**• The coldest month (** Fig. 4, Fig. 5, Fig. 6 **)**

Table 1 above provides a statistical summary of the monthly data. It shows the averages, standard deviations, maximums and minimums of the microclimatic parameters of different experimental environments, namely: open room S1 (TS1, HS1, DS1), closed uninhabited room S2 (TS2, HS2, DS2), inhabited room S3 (TS3, HS3, DS3) and outside EX (TEX, HEX, DEX).

**Table 1:** Summary statistics.

Environment		S1			S2			S3			EX		
Months	Statistics	TS1	HS1	DS1	TS2	HS2	DS2	TS3	HS3	DS3	TEX	HEX	DEX
January	Means	28,8	80,1	24,9	29,6	87,2	27,2	29,5	82,3	26,1	29,8	77,8	25,0
	Standard deviation	1,5	5,9	0,7	1,1	2,5	1,0	1,1	3,3	0,8	4,0	15,6	0,8
	Max	32,0	89,9	27,4	32,3	92,3	29,7	32,0	88,6	28,1	39,4	98,8	27,4
	Min	24,6	56,9	22,0	26,4	72,5	23,9	26,3	59,1	22,4	23,3	39,0	20,4
February	Means	29,5	78,7	25,2	30,2	82,2	26,7	30,3	78,6	26,1	30,8	75,7	25,4
	Standard deviation	1,8	6,6	0,8	1,3	3,4	0,9	1,3	3,7	0,9	4,3	16,2	0,8
	Max	33,2	90,6	27,0	32,6	89,9	28,4	32,7	87,2	28,3	41,6	99,6	28,1
	Min	24,3	53,4	21,5	26,1	73,5	23,4	25,9	66,1	22,7	23,5	33,6	19,9
March	Means	28,6	80,3	24,8	29,2	83,2	25,9	29,6	79,6	25,6	29,9	75,9	25,0
	Standard deviation	2,2	6,2	1,2	1,5	2,7	1,2	1,6	3,1	1,2	2,7	8,4	1,2
	Max	33,8	93,2	27,5	32,9	90,5	28,3	33,3	88,2	28,4	36,9	93,5	27,7
	Min	24,0	58,0	21,2	25,3	73,5	22,8	25,9	69,9	22,6	24,3	50,9	21,1
April	Means	29,2	79,7	25,2	29,7	82,4	26,3	29,8	79,3	25,8	30,4	75,8	25,3
	Standard deviation	2,2	6,3	1,2	1,5	2,7	1,2	1,6	3,1	1,2	3,3	10,7	1,2
	Max	34,8	91,1	27,3	32,9	88,5	28,8	33,4	86,7	29,3	40,0	93,6	28,0
	Min	24,6	60,4	21,8	26,3	72,9	23,3	25,8	67,9	21,9	23,3	44,6	21,8
May	Means	28,8	79,4	24,8	29,2	82,4	25,8	29,4	79,1	25,3	30,1	75,0	24,9
	Standard deviation	2,3	6,7	1,1	1,5	3,0	1,1	1,5	3,6	1,1	3,0	9,4	1,1
	Max	34,8	92,6	27,4	32,4	89,7	28,4	32,6	87,7	27,7	37,5	91,7	27,8
	Min	24,3	58,7	22,1	25,6	71,1	23,2	25,8	65,6	22,6	24,6	50,3	21,8
Jun	Means	27,4	84,6	24,5	27,8	87,0	25,4	28,2	84,5	25,3	28,4	81,2	24,7
	Standard deviation	1,8	5,9	0,8	1,3	3,0	1,0	1,3	3,6	0,8	2,5	8,6	0,8
	Max	33,3	96,1	26,6	31,4	94,0	27,9	31,7	92,6	27,1	36,4	95,8	27,3
	Min	23,7	63,0	21,8	24,8	78,8	22,5	25,1	73,0	22,8	23,6	55,3	21,8
July	Means	26,2	88,9	24,1	26,6	93,2	25,3	26,9	89,8	25,1	27,2	85,6	24,4
	Standard deviation	1,4	4,8	0,7	0,9	1,4	0,8	1,0	2,3	0,7	1,9	7,0	0,7
	Max	31,3	95,8	26,8	30,0	97,1	27,8	30,0	94,8	27,4	34,7	96,5	26,7
	Min	23,4	69,0	22,0	24,7	84,9	23,6	24,7	80,4	23,1	23,7	59,4	21,9
August	Means	25,1	92,7	23,8	25,6	96,5	25,0	25,9	93,3	24,6	26,1	89,1	24,1
	Standard deviation	1,2	3,1	0,7	0,9	0,9	0,9	0,9	1,5	0,8	1,7	5,6	0,7
	Max	29,4	96,7	26,3	29,0	98,1	27,4	28,7	96,0	26,9	32,4	95,6	26,7
	Min	23,0	77,0	22,1	24,0	87,8	23,2	24,1	86,2	23,0	23,3	66,0	22,5
September	Means	26,7	87,8	24,4	27,2	94,9	26,3	27,5	90,9	25,8	27,9	83,1	24,6
	Standard deviation	1,6	5,4	0,7	1,1	1,5	1,0	1,2	3,1	0,9	2,2	8,2	0,7
	Max	31,3	96,6	26,8	30,7	98,0	29,3	30,4	96,6	29,0	34,7	95,7	26,8
	Min	23,4	68,1	22,0	24,6	83,1	23,9	24,6	79,2	23,4	23,7	56,1	21,6
October	Means	26,9	85,7	24,3	27,6	92,1	26,2	28,0	87,2	25,6	27,9	81,8	24,3
	Standard deviation	1,7	5,2	0,8	1,2	2,0	1,1	1,3	3,4	0,9	2,5	8,7	0,8
	Max	32,3	95,1	26,5	31,5	96,6	29,1	31,9	93,5	27,8	35,8	94,9	26,6
	Min	23,8	67,9	22,4	24,9	80,5	23,9	25,2	74,0	23,5	23,9	55,1	22,2
November	Means	27,7	84,9	24,8	28,6	88,0	26,3	28,8	83,8	25,8	28,7	80,8	24,9
	Standard deviation	1,9	5,1	1,1	1,6	2,3	1,3	1,6	3,7	1,0	2,6	8,1	1,1
	Max	32,3	94,2	27,1	32,4	93,4	29,3	32,5	92,1	28,5	35,5	95,6	27,4
	Min	22,7	64,8	21,5	24,1	81,8	22,4	24,5	70,6	22,8	22,7	53,3	21,7
December	Means	29,1	80,9	25,4	30,2	82,4	26,8	30,3	79,1	26,1	30,2	76,7	25,5
	Standard deviation	1,6	5,7	0,7	1,2	3,4	0,9	1,3	3,9	0,8	2,2	8,3	0,7
	Max	32,6	91,7	26,8	32,7	90,6	28,9	32,9	87,5	28,4	35,7	90,0	27,1
	Min	25,7	65,0	22,1	27,6	73,8	23,8	27,3	67,8	23,2	26,0	54,3	22,1

## 4 Experimental Design, Materials and Methods

### 4.1 Experimental area

**Fig. 7:**
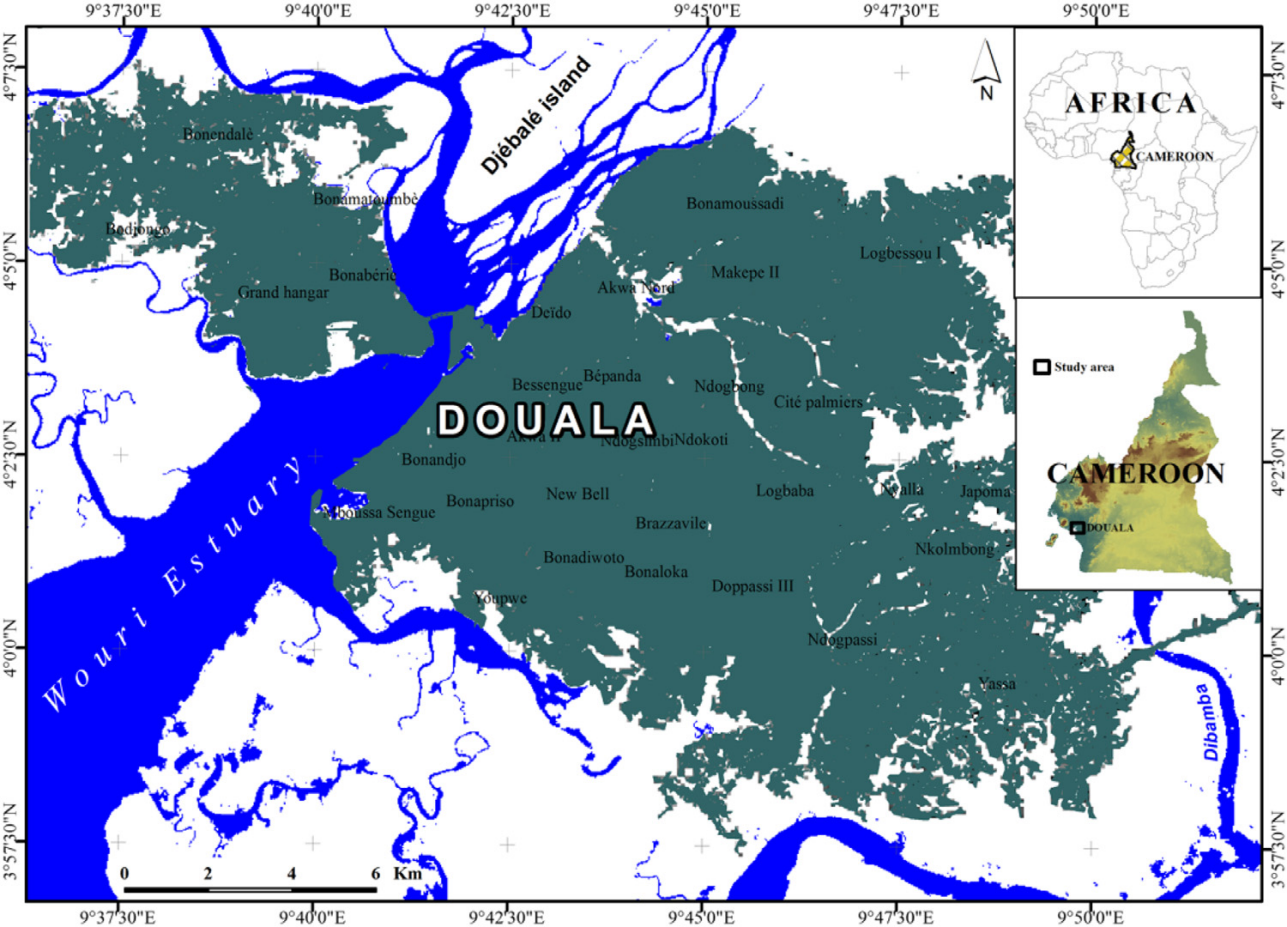
Study area [4].

The experiment took place in Douala (Fig. 7), a coastal city in Cameroon, a country in Central Africa. Douala is located in a hot and humid area [1,2]. Temperatures range from 19 °C to 32 °C with an average of 25 °C and the humidity varies between 65 % and 98 % due to its proximity to the Atlantic Ocean. Rainfall is abundant; there are 175 to 200 days of rain per year and approximately of 4500 mm of rainfall. Housing is haphazard in the outlying areas and built from breeze blocks [3].

### 4.2 Experimental building

**Fig. 8:**
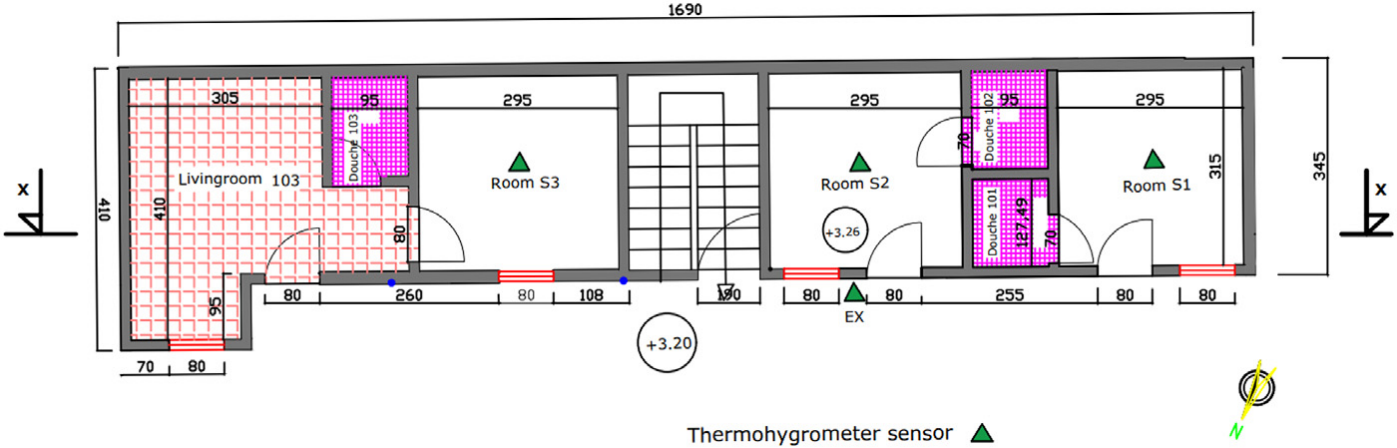
Experimental building plan.

**Fig. 9:**
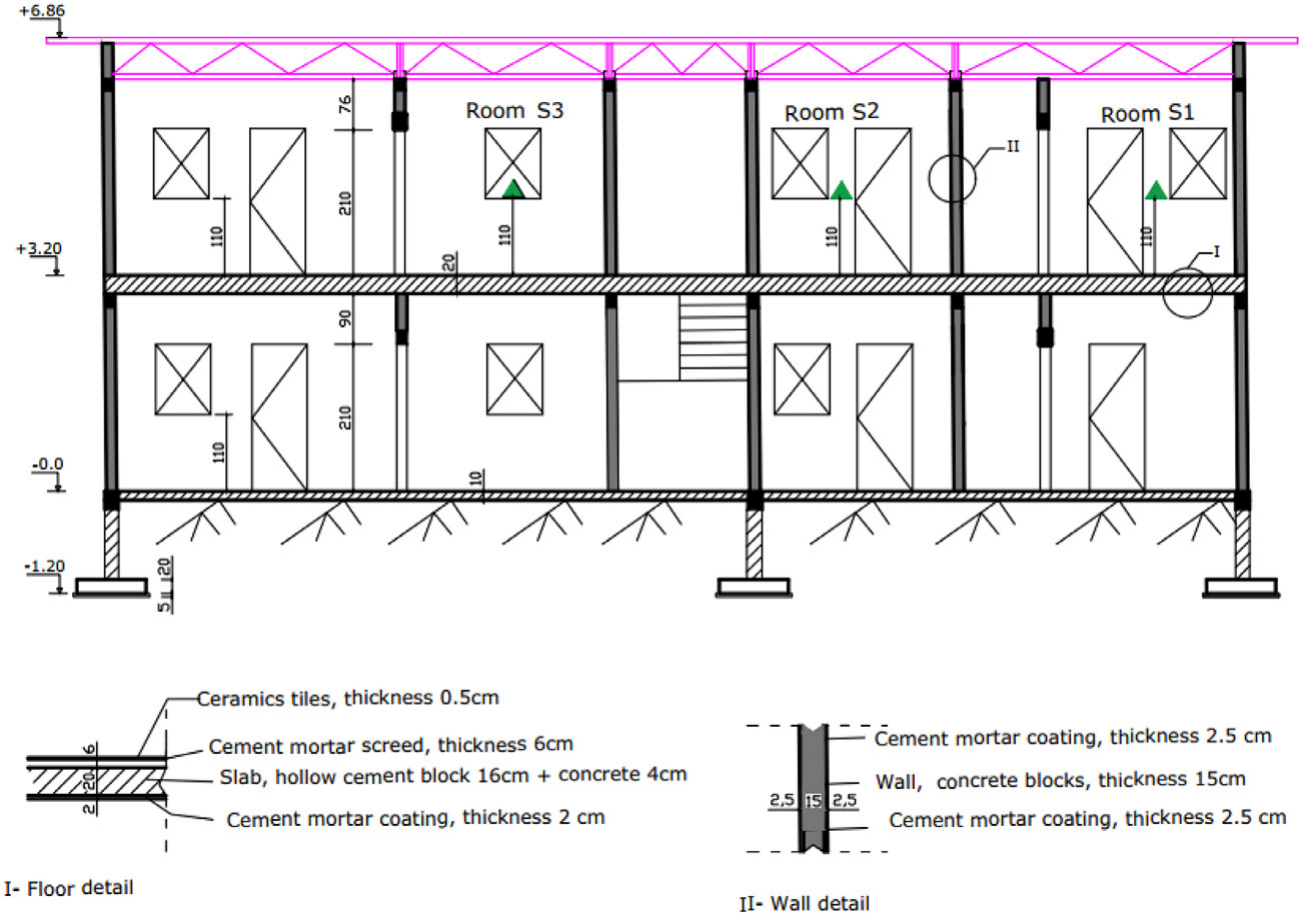
Longitudinal section.

**Fig. 10:**
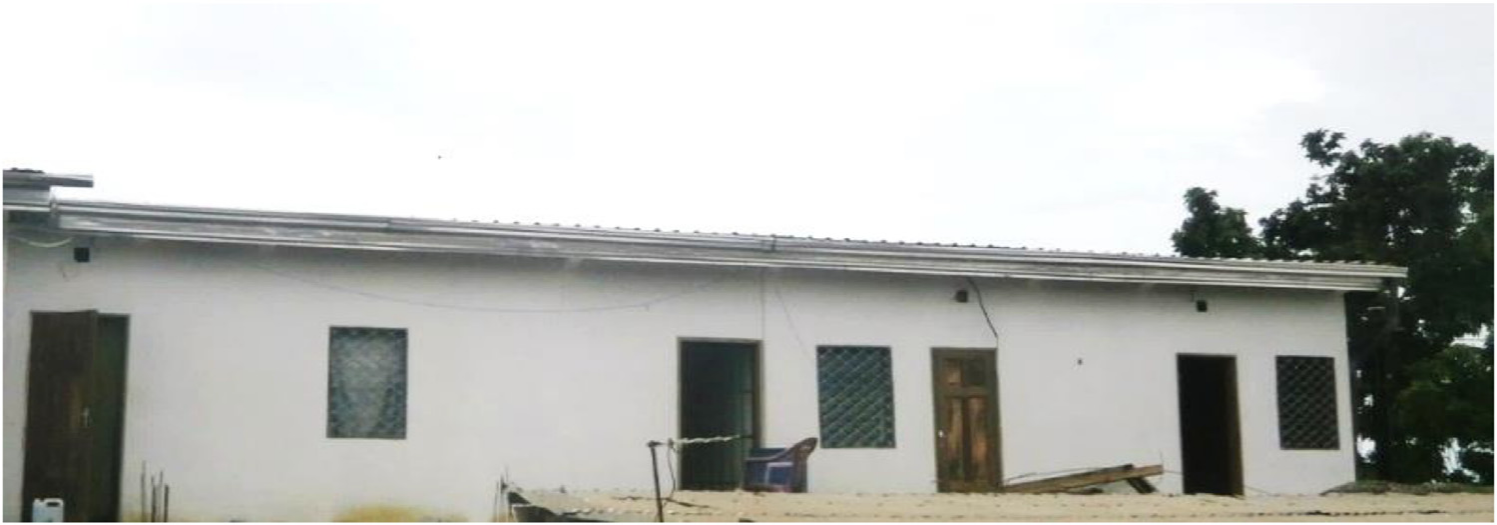
The experimental building.

The Figs. 8 and 9 show the plan and longitudinal section of the three experimental rooms. Fig. 10 is a photograph of the experimental building. The walls are built of 15 cm thick concrete blocks, covered with 2.5 cm of cement plaster on each side. The floor is a slab covered with a 6 cm thick screed and 0.5 cm thick ceramic tiles. The roof is made of 0.5 mm aluminum sheet, with a 4 mm thick plywood ceiling. The ceiling height is 2.80 m.

In this regard, data acquisition equipment is located in the experimental rooms and outside:

- Room S1 open and unoccupied: door and window open;
- Room S2 unoccupied: wooden door and window closed;
- Room S3 occupied by one (01) person. This person was a worker who left at 7 a.m. and returned in the evening around 8 pm. With a 75 W fan and a 15 W lamp, this person rarely received visitors.
- The outside atmosphere (EX).

### 4.3 Data acquisition equipment

Data acquisition equipment consists of four thermohygrometers and a computer.

**Fig. 11:**
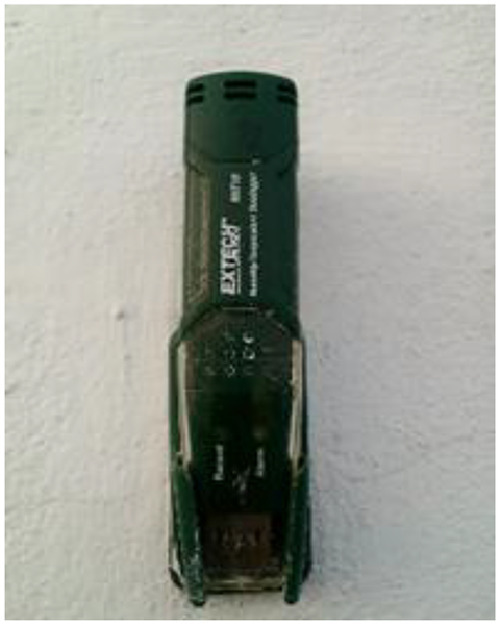
Thermohygrometer recorder.

These are EXTECH RHT10 relative humidity and temperature data loggers, shown in Fig. 11 below.

This recorder can simultaneously measure and record up to 16,000 relative humidity data points in the range of 0 to 100% ± 5.0 and 16,000 temperature data points in range of −35 to 80 °C.

The sensor specifications are shown in Table 2 below.

**Table 2:** Characteristics of the thermo-hygrometer.

Relative Humidity (%)	General amplitude	0 ⇒ 100
	Accuracy (0 ⇒ 20 and 80 ⇒ 100)	± 5.0
	Accuracy (20 ⇒ 40 and 60 ⇒ 80)	± 3 0.5
	Accuracy (40 ⇒ 60)	±3 0.0
Temperature (°C)	General amplitude	- 40 à 70
	Accuracy (−40 ⇒ −10 and + 40 ⇒ + 70)	± 2
	Accuracy (−10 ⇒ 40)	±1
Download rate from 2 s to 24 h	Selectable sampling interval:	

### 4.4 Computer

A laptop computer is used for data backup and processing.

Data acquisition with thermohygrometers

In each of the three rooms (S1, S2 and S3), thermohygrometers are suspended from the ceiling, 1.10 m above the floor, in accordance with the ISO7726 (1998) [5] relating to instruments for measuring thermal comfort parameters in buildings. In the outdoor environment “EX”, another thermohygrometer is placed to acquire the temperature, relative humidity of the outside air and dew point. The EXTECH^Ⓡ^ RHT10 sensor takes 16,000 temperature measurements and 16,000 humidity measurements. Selectable data sampling frequencies: 2 s, 5 s, 10 s, 30 s, 1 m, 5 m, 10 m, 30 m, 1 hr, 2 hr, 3 hr, 6 hr, 12 hr, 24 hr. However, the recording interval is set to one (01) minute.

## Limitations

There are limitations due to the fact that variations in the number of occupants were not taken into account during friendly visits to the occupied room S3. Furthermore, the duration of use of the lamp, fan, and other potential heat sources was not taken into account. In addition, data collection was not extended to other sites. The sensor's internal algorithm was not accessible.

## Ethics Statement

The authors have read and follow the ethical requirements for publication in Data in Brief and confirming that the current work does not involve human subjects, animal experiments, or any data collected from social media platforms.

## CRediT Author Statement

Thomas Janvier Matongo funding acquisition, resources and writing, data Curation - original draft; Gilbert Roméo Hubert Ngock investigation, formal analysis and validation; Emmanuel Yamb project administration; Jean Gaston Tamba conceptualization and supervision.

## Data Availability

Mendeley DataFigures of Data on air temperature, relative humidity, and dew point in three housing modes in a building in a hot and humid area of Douala, Cameroon (Original data). Mendeley DataData on air temperature, relative humidity, and dew point in three housing modes in a building in a hot and humid area of Douala, Cameroon (Original data).
